# Integration of thermal energy storage for sustainable energy hubs in the forest industry: A comprehensive analysis of cost, thermodynamic efficiency, and availability

**DOI:** 10.1016/j.heliyon.2024.e36519

**Published:** 2024-08-19

**Authors:** Behnam Talebjedi, Timo Laukkanen, Henrik Holmberg

**Affiliations:** Department of Mechanical Engineering, School of Engineering, Aalto University, Espoo, Finland

**Keywords:** Thermal energy storage (TES), Phase change material, Reliability &availability analysis, Markov process, Long-short-term memory (LSTM), Energy hubs (EH), Gated recurrent unit (GRU)

## Abstract

Thermal energy storage (TES) offers a practical solution for reducing industrial operation costs by load-shifting heat demands within industrial processes. In the integrated Thermomechanical pulping process, TES systems within the Energy Hub can provide heat for the paper machine, aiming to minimize electricity costs during peak hours. This strategic use of TES technology ensures more cost-effective and efficient energy consumption management, leading to overall operational savings. This research presents a novel method for optimizing the design and operation of an Energy Hub with TES in the forest industry. The proposed approach for the optimal design involves a comprehensive analysis of the dynamic efficiency, reliability, and availability of system components. The Energy Hub comprises energy conversion technologies such as an electric boiler and a steam generator heat pump. The study examines how the reliability of the industrial Energy Hub system affects operational costs and analyzes the impact of the maximum capacities of its components on system reliability. The method identifies the optimal design point for maximizing system reliability benefits. To optimize the TES system's charging/discharging schedule, an advanced predictive method using time series prediction models, including LSTM (Long Short-Term Memory) and GRU (Gated Recurrent Unit), has been developed to forecast average daily electricity prices. The results highlight significant benefits from the optimal operation of TES integrated with Energy Hubs, demonstrating a 4.5–6 percent reduction in system operation costs depending on the reference year. Optimizing the Energy Hub design improves system availability, reducing operation costs due to unsupplied demand penalty costs. The system's peak availability can reach 98 %, with a maximum heat pump capacity of 2 MW and an electric boiler capacity of 3.4 MW. The GRU method showed superior accuracy in predicting electricity prices compared to LSTM, indicating its potential as a reliable electricity price predictor within the system.

## Highlights

• A novel method for optimal design and operation of an energy hub in the forest industry.
• The study examines system robustness, reliability, and cost efficiency.
• Analyzed the link between the system operation cost and reliability.
• Time-series models predict daily market electricity prices for optimal TES operations.

**Table:** Nomenclature

EH	Energy Hub	HSC	Heat supply capacity
PCM	Phase change material	HD	Heat demand
TES	Thermal energy storage	SA	System availability (%)
LHTES	Latent heat thermal energy storage	BP	backpropagation
TMP	Thermomechanical pulping	ReLU	Rectified Linear Unit
PM	Paper machine	EP	Market electricity price (€/MWh)
SGHP	Steam generator heat pump	OC	Operation cost (€)
HP	Heat pump	AF	Annuity factor
EB	Electric boiler	TC	Total cost
RL	Refining line	RMSE	Root mean square error
SSM	State-space method	R	Correlation coefficient
RNN	Recurrent Neural Network	λ	Failure rate
LSTM	Long-short term memory	μ	Repair rate
GRU	Gated recurrent units	p	Limiting state probability

## 1 Introduction

Nowadays, the imperative for industries to enhance profitability and maintain competitiveness in the market has driven a keen focus on reducing operation costs. In energy-intensive sectors like pulp and paper industries, operation cost reduction becomes particularly crucial, and innovative strategies are being employed, notably in the scope of energy cost savings. Thermomechanical pulping (TMP) is a pulp-making process that involves mechanically grinding wood chips at high temperatures, often with steam. This method is advantageous because it efficiently breaks down wood fibers, resulting in a high yield of pulp suitable for paper production while minimizing the need for chemical additives [1]. The thermomechanical pulping process is highly electricity-intensive and generates substantial heat during refining. However, paper mills necessitate a significant amount of heat for the drying process. In integrated pulp and paper mills, where TMP and paper production coexist on the same site, optimizing energy utilization becomes paramount. A noteworthy approach involves harnessing the excess heat generated by the TMP process through a heat recovery unit, supplementing the paper mill's thermal requirements [2]. Additionally, the utility section, functioning as an energy hub, plays a paramount role by providing supplementary heat to the paper mill. In the pursuit of further efficiency gains, the integration of thermal energy storage technologies emerges as a progressive and promising solution, mitigating energy costs by strategically managing electricity consumption during peak electricity pricing periods.

Thermal energy storage systems enhance energy efficiency by storing and utilizing excess thermal energy during peak demand, reducing energy waste and costs. TES supports peak load management by shifting grid electricity use from peak to off-peak hours, alleviating stress on the grid and lowering reliance on expensive power generation. Furthermore, TES aids renewable energy integration, lowers environmental impact, and enhances grid stability, offering cost savings, demand response opportunities, and improved energy security across various applications [3]. Tawalbeh et al. [4] conducted an extensive review of the latest developments in materials used for thermal energy storage applications. Miró et al. [5] delivered an in-depth analysis focusing on the adoption of thermal energy storage systems for the recovery of waste heat in industrial settings. Opolot et al. [6] conducted a review to highlight the key considerations, challenges, cost analysis, and performance metrics associated with integrating latent heat thermal energy storage (LHTES) systems, particularly those using phase change materials, in various applications. Davoudi et al. [7] presented a peer-to-peer thermal energy transaction framework for small-scale heat producers and consumers, allowing them to act as price makers. It optimizes their participation in heat and electricity markets, demonstrating its effectiveness through numerical results. Calderon et al.'s [8] bibliometric analysis reveals an increasing interest in thermal energy storage within the scientific sector from 2010 onwards. According to their research, most scientific investigations concentrate on latent heat thermal energy storage, with research activities showing exponential growth over the past twenty years. A recent study [9] presents the most recent progress in research and development concerning latent heat thermal energy storage. Cirocco et al. [10] studied the implementation of a latent heat thermal energy storage system with advanced control and forecast algorithms in an industrial food processing plant, utilizing onsite PV and grid electricity for refrigeration. The integrated system effectively manages electricity demand, resulting in a net estimated cost savings of 10,435 AUD over ten months, with the potential for annual savings of 48,700 AUD by doubling the thermal energy storage capacity. Barnetche et al. [11] evaluated two methods of integrating latent storage into solar heating and cooling systems for industrial processes. They discovered that connecting thermal energy storage in series is the most efficient and economical option leading to reductions in operational and overall costs.

This paper investigates the integration of an energy hub featuring thermal heat storage (PCM material), an electric boiler, and a steam generator heat pump with the TMP mill and paper machine. The novel approach proposed relies on assessing the reliability and availability of system components integrated with thermodynamic and cost analyses. The modeling of thermal energy storage operation relies on a thorough and precise physical representation of the phase change material (PCM) utilized as the storage medium. The primary goal is to minimize the operational costs of the energy hub through a multifaceted approach. The utilization of thermal energy storage emerges as a crucial strategy to limit electricity expenses during peak electricity pricing periods. Additionally, the paper adopts a Markov chain approach to assess and enhance the availability of the system, strategically avoiding extra unsupplied demand penalty costs. The unsupplied demand penalty cost represents the financial compensation owed by the energy hub operator to the energy customer for each kilowatt-hour (KWh) shortage in providing the heat demand. By comprehensively examining these factors, this study aims to develop an efficient and cost-effective energy hub solution that not only addresses peak electricity pricing challenges but also ensures the system's reliability, thereby mitigating potential unsupplied demand penalty costs.

To develop an effective optimization strategy for maximizing the efficiency of a thermal energy system within the proposed energy hub configuration, it is imperative to incorporate accurate electricity load predictions [12]. These predictions serve as a crucial foundation for optimal scheduling, facilitating the efficient charging and discharging of thermal energy storage. However, as renewable energy becomes a larger proportion of the power grid, the variability in power generation also rises significantly, making it challenging to forecast electricity prices accurately [13]. Current approaches to forecasting electricity prices can be categorized into three main groups: conventional techniques, machine learning approaches, and deep learning methodologies [14]. Traditional forecasting methods fall within the first category and comprise models like autoregressive integrated moving average (ARIMA) [15] and generalized autoregressive conditional heteroskedasticity (GARCH) [16], along with enhanced versions such as autoregressive moving average exogenous (ARMAX) [17]. Nevertheless, traditional methods necessitate significant data stability. Machine learning techniques encompass support vector machine (SVM), random forest (RF), and the back propagation neural network [18], among others. Despite yielding improved prediction outcomes attributed to their heightened robustness and nonlinear mapping capacities in contrast to traditional methods, they still exhibit limitations in probing the internal temporal dynamics of time series data. Hence, deep learning algorithms tailored for sequence modeling have arisen, incorporating standard models like recurrent neural networks (RNNs) and their variations such as long short-term memory (LSTM) and the gate recurrent unit (GRU) [19]. The design of these models is proficient in handling continuous sequences, making them highly useful in the present domain of electricity price forecasting. A Gated Recurrent Unit (GRU) and Long-short-term memory (LSTM) networks have demonstrated efficiency in modeling and forecasting time series data [20]. They excel over other time series AI models because they can better capture and retain long-term dependencies in sequential data [21]. The GRU's gating mechanisms enable efficient handling of sequential data by managing memory and mitigating vanishing gradient issues, enhancing learning accuracy and efficiency. Selecting the right architecture involves considering data characteristics, computational resources, and model complexity, with experiments necessary to determine the optimal solution for a specific task.

Long-short-term memory networks are popular for their ability to effectively process, predict, and classify complex and non-linear time series data [22]. The performance of an LSTM algorithm was evaluated using a publicly accessible dataset of residential meters, as referenced in Ref. [23]. The outcomes demonstrated that, in short-term residential load forecasting, the LSTM algorithm surpassed competing machine learning (ML) algorithms. Taheri et al. [24] investigated the forecasting of electricity demand time series by employing a hybrid prediction model that combines LSTM with empirical mode decomposition (EMD). The EMD algorithm breaks down the load time series data into intrinsic mode functions (IMFs), and distinct LSTM models are created for each IMF. These individual LSTM models' outputs are then aggregated into a meta-learner for the final energy demand prediction. Through experiments conducted on the California ISO dataset, their hybrid model demonstrated superior performance compared to single LSTM, LR, and XGBoost algorithms. Xiong et al. [25] proposed a new data-driven method that incorporates flow rates into the modeling of Vanadium Redox Flow Batteries (VRB), aiming to improve data processing and prediction accuracy for VRB behaviors. Using GRU's excellent time series processing, the model accurately predicts terminal voltage with a minimal error margin of 0.023 V (1.3 %) across various operating ranges. In this study, both GRU and LSTM algorithms were utilized to enhance market electricity price prediction accuracy, contributing to optimal decision-making for scheduling thermal energy storage charging and discharging. To the best of the authors' knowledge, the utilization of the GRU algorithm for forecasting daily average electricity prices has not been thoroughly investigated in the literature for practical real-world applications. Therefore, in this research, the GRU model has been studied to predict the daily average electricity price.

This paper explores the integration of an energy hub, which includes thermal heat storage using PCM material, an electric boiler, and a steam generator heat pump, with the TMP mill and paper machine. While significant research has been dedicated to enhancing the thermal density and longevity of thermal energy storage through material studies, the operational dynamics of these storage systems and their integration into energy systems, such as energy hubs, have traditionally received limited attention in prior studies. By integrating reliability and availability analyses alongside thermodynamic and cost analyses, this method offers a comprehensive framework for optimizing EH-TES systems, ensuring both efficiency and resilience in industrial operations. This innovative approach not only enhances the understanding of TES integration but also provides actionable insights for maximizing cost-effectiveness and system performance within the forest industry context. The foremost paper's novelty and contribution are outlined as follows:

• A novel method has been proposed for the optimal design and operation of an energy hub integrated with thermal energy storage (TES) in the forest industry. The energy hub's proposed optimal design approach is based on a thorough analysis of the dynamic efficiency, reliability, and availability of the system components.
• Employing an innovative methodology, the study investigates the relationship between operational expenses and system robustness, providing insights for enhancing both cost efficiency and reliability. The correlation between system operation cost and reliability has been thoroughly analyzed, providing insight into how the reliability of a system could be influenced by the maximum capacities of its energy conversion components.
• The developed optimal operation strategy incorporates advanced time-series prediction models to precisely forecast the average market electricity price. These models, developed specifically for integration with the EH system, enable optimal charging and discharging of thermal energy storage, thus reducing operational costs.

## 2 Thermal energy storage

Thermal Energy Storage (TES) has gained significance in thermodynamic systems due to the variability of solar radiation and intermittent energy availability. These systems are commonly classified into three groups based on their methods of storing thermal energy. Sensible heat storage systems depend on elevating and lowering the temperature of a storage medium, such as water. Latent heat storage systems make use of the thermal energy associated with phase changes, such as the melting and crystallization of a substance. Thermochemical energy storage, stores thermal energy through reversible reactions, such as water vapor sorption on a material's surface. Table 1, as presented in Ref. [26], provides standard metrics for TES systems. The table categorizes thermal energy storage (TES) systems into three types: Sensible (Hot Water), Phase Change Material (PCM), and Chemical Reactions. For each TES type, it provides key metrics such as energy storage capacity in kilowatt-hours per ton (kWh/t), power output range in megawatts (MW), efficiency as a percentage, and cost in euros per kilowatt-hour (€/kWh). Sensible storage systems, which use hot water, have a stage period ranging from days to months, indicating their suitability for long-term energy storage. PCM systems, which leverage materials that change phases, offer flexibility with stage periods ranging from hours to months, making them viable for both short and extended storage durations. Chemical reaction-based systems have the shortest stage periods, from hours to days, suitable for short-term energy storage needs.

**Table 1:** Characteristics of various TES materials.

TES type	Capacity (kWh/t)	Power (MW)	Efficiency (%)	Stage Period	Cost (€/kWh)
Sensible (Hot Water)	10–50	0.001–10.0	50–90	days/months	0.1–10
Phase Change Material (PCM)	50–150	0.001–1.0	75–90	hours/months	10–50
Chemical reactions	120–250	0.01–1.0	75–100	hours/days	8–100

The stage period is a critical factor as it indicates how long the TES system can maintain and release stored energy effectively. Sensible (Hot Water) systems are best for long-term storage due to their extended stage periods, while PCM systems provide versatility across various storage durations. Chemical Reaction systems are optimized for shorter-term storage, useful in scenarios requiring rapid energy deployment. The cost values, denoted in €/kWh, provide a comparative measure of the expense associated with storing 1 kW-hour of energy using each type of TES, aiding in evaluating the economic feasibility of different storage technologies. In any storage system, having a high-energy storage density and robust power capacity for both charging and discharging is highly advantageous. It's widely recognized that there are three established TES methods spanning a temperature range from −40 °C to over 400 °C: sensible heat storage, latent heat storage linked with phase-change materials (PCMs), and thermo-chemical heat storage involving chemical reactions [23].

For industrial applications, latent heat storage proves to be a remarkably effective thermal energy storage technology, surpassing other alternatives. This superiority lies in its capacity to store and release substantial amounts of energy during phase transitions, such as solidification and melting of phase-change materials (PCMs). The key advantage is the isothermal nature of these phase transitions, where the temperature remains nearly constant during the energy exchange, enabling a more stable and efficient thermal performance. In addition, the high energy density associated with latent heat storage allows for compact system designs, ideal for industrial settings with space constraints. These characteristics make latent heat storage a top choice for industrial applications where the efficient management of thermal energy is paramount.

### 2.1 Latent-heat storage (LHS)

LHS materials, also referred to as phase change materials (PCMs), are recognized for their ability to release or absorb energy when their physical state changes [27]. The primary energy storage occurs during the phase-change transition, typically at a consistent temperature, and is directly related to the material's latent heat. Employing a latent heat storage system with PCMs proves to be an efficient method for storing thermal energy, offering benefits such as high-energy storage capacity and a storage process that maintains a constant temperature [28]. The primary benefit of using latent heat storage instead of sensible heat storage (SHS) lies in its ability to store heat within a nearly uniform temperature range. In the beginning, these substances behave much like SHS materials, with temperature increasing linearly alongside the system's enthalpy. Nonetheless, as the process continues, heat is either absorbed or released at nearly a constant temperature when a change in physical state occurs.

Latent heat storage relies on the absorption or release of heat during the transition of a storage material between solid and liquid, liquid and gas, or vice versa. The storage capacity denoted as $Q_{s}$ and measured in joules, for an LHS system utilizing a phase change material (PCM) is determined by the equation below, as detailed in Ref. [29].

(1) $$Q_{s}=\int_{t_{i}}^{t_{m}}mc_{p}dt+m_{t}\Delta q+\int_{t_{m}}^{t_{f}}mc_{p}dt$$

(2) $$Q_{s}=m\left[c_{ps}\left(t_{m}-t_{i}\right)+f\Delta q+c_{pl}\left(t_{f}-t_{m}\right)\right]$$

$t_{m}$ represents the temperature at which the phase change occurs, measured in degrees Celsius (°C). m is the mass of the phase change material medium, measured in kilograms (kg). ${cp}_{s}$ stands for the average specific heat of the solid phase between $t_{i}$ and $t_{m}$, measured in kilojoules per kilogram per Kelvin (kJ/(kg·K)). $c_{pl}$ represents the average specific heat of the liquid phase between $t_{m}$ and $t_{f}$, measured in joules per kilogram per Kelvin (J/(kg·K)). f signifies the proportion of the material that has melted, often referred to as the melt fraction. Δq denotes the latent heat of fusion, measured in joules per kilogram (J/kg). For instance, Glauber's salt (${\text{Na}}_{2}{\text{SO}}_{4}.10H_{2}O)$ exhibits the following properties: $c_{ps}$ is approximately 1950 J/(kg·°C), $c_{pl}$ is roughly 3550 J/(kg·°C), and Δq equals 2.43 × $10^{5}$ J/kg at a temperature of 34 °C.

## 3 The fundamental concept of a paper machine

**Fig. 1:**
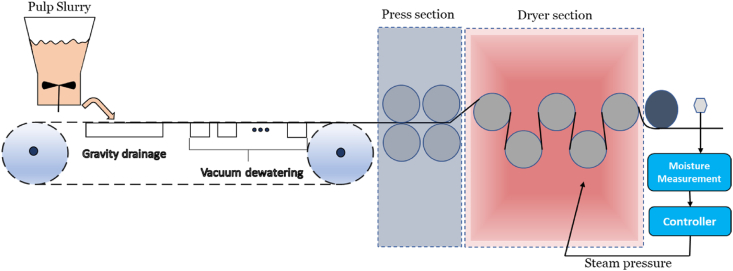
A visual representation of a paper machine's structural design, including a drying section equipped with multiple cylinders.

Fig. 1 illustrates a schematic representation of a paper machine. During the forming stage, the slurry is evenly spread across a moving perforated screen known as the wire. Dewatering primarily takes place through the force of gravity in this section of the paper machine, often referred to as the wire or forming section. By the end of this section, a continuous web with a dry solids content ranging from 15 % to 25 % is produced. The web then progresses into the press section.

In the press section, mechanical compression is employed to reduce the water content to a solid level that typically falls between 33 % and 55 %. The exact level depends on the type of paper being produced and the design of the press section. The third segment of the paper machine is known as the drying or dryer section. In this section, the paper web travels overheated, rotating cast iron cylinders, and most of the remaining water is removed through evaporation. As the paper exits the dryer section, its solid content has increased to approximately 90–95 %. Dewatering in the dryer section relies solely on thermal energy, and the application of heat transfer is crucial during this stage of the papermaking process [30]. The dryer section of the paper machine is an ideal area where thermal energy storage can be integrated. This integration allows the stored thermal energy to provide the necessary heat for the drying process of the paper machine. The heat required for the drying section of the paper machine must be consistently provided. To ensure the reliability of the system, it is necessary to have an additional heat supply alongside the thermal energy storage (TES). This supplementary heat source acts as a backup, guaranteeing that the drying process remains uninterrupted and effective, even if the TES cannot meet the demand alone.

## 4 RNN-like time series model development

This section presents a comprehensive examination of two distinct RNN-like models, specifically the Gated Recurrent Unit (GRU) and the Stacked Long Short-Term Memory (LSTM), developed and evaluated for the precise prediction of average daily electricity prices. The comparative analysis encompasses the architectural details, training methodologies, and performance metrics. Accurate electricity forecasting plays a key role in optimizing the scheduling of thermal energy storage systems for charging and discharging operations. This critical aspect ensures efficient management of energy resources, enhancing overall system performance and contributing to the sustainable and reliable operation of the energy storage infrastructure.

GRU and LSTM are both types of recurrent neural network (RNN) architectures used in the context of time series modeling. While they share some similarities, they also have key differences in their structures. In essence, both GRU and LSTM are designed to address the vanishing gradient problem, which is a common issue in training traditional RNNs on long sequences. They achieve this by incorporating gating mechanisms that allow the networks to selectively retain and forget information from previous time steps.

### 4.1 Gated recurrent unit (GRU) neural network

Typical neural networks assume that input and output variables are independent of each other and do not consider the impact of prior information on inputs, which is not suitable for predicting long-term time series. However, RNNs can recall previous information and incorporate it to predict the target. In general, RNNs excel at retaining past information, enabling them to effectively process information in arbitrarily long sequences. Nevertheless, conventional RNNs using the backpropagation (BP) training algorithm struggle with learning long-term dependencies, resulting in practical weaknesses and modeling challenges.

**Fig. 2:**
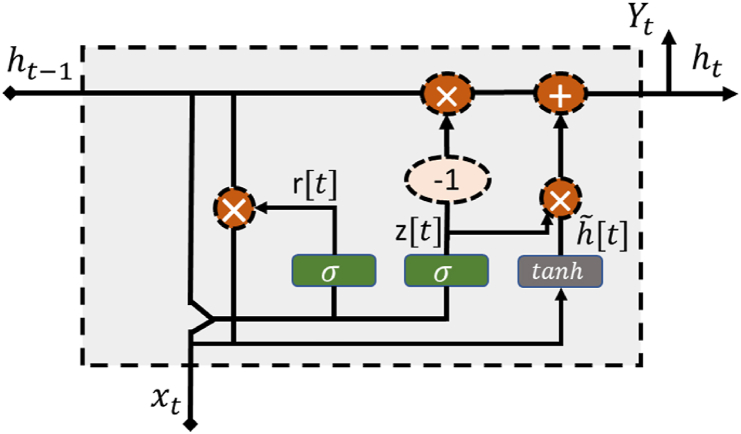
Internal operations of a GRU cell.

GRU utilizes a distinctive gating mechanism within recurrent neural networks to address inherent shortcomings and enhance the ability to learn long-term dependencies, specifically addressing issues like vanishing gradients observed in traditional RNNs. To overcome these challenges, GRU incorporates a specific structure into its cell. Fig. 2 elucidates the functional mechanism and internal memory cell of GRU. In this depiction, $r_{t}$ represents the reset gate of GRU at time t, $x_{t}$ denotes the input at time t, and $h_{t-1}$ signifies the hidden state at time t−1. Additionally, " $W_{r}$" and " $U_{r}$" correspond to the weight matrices for input data and hidden states, respectively.

In contrast to LSTM, GRU does not feature isolated memory cells. Instead, it employs a single hidden state $h_{t}$ to propagate information across different time steps. Furthermore, GRU combines the input and forget gates into an update gate (z), and the reset gate ($r_{t}$) is directly applied to $h_{t-1}$ to obtain $h_{t}$ (the candidate state), which is then added to $h_{t}$ [31]. The paradigm of GRU is elucidated through the following equations.

(3) $$z_{t}=\sigma \left(W_{z}x_{t}+U_{z}h_{t-1}+b_{z}\right)$$

(4) $$r_{t}=\sigma \left(W_{r}x_{t}+U_{r}h_{t-1}+b_{r}\right)$$

(5) $${\tilde{h}}_{t}=\tan\;h\left(W\tilde{h}x_{t}+U\tilde{h}\left(r_{t}\times h_{t-1}\right)+b\tilde{h}\right)$$

(6) $$h_{t}=\left(1-z_{t}\right)\times h_{t-1}+z_{t}\times {\tilde{h}}_{t}$$

In the provided equations, σ and tanh represent the logistic sigmoid and hyperbolic tangent functions, respectively. The symbol × denotes element-wise multiplication, and b represents the bias vector. All these components constitute learnable and non-shared factor sets. Due to the influence of the sigmoid function, the gates in the system are vectors with values constrained within the range (0, 1). When the reset gate $r_{t}$ is closed, GRU disregards $h_{t-1}$ and is influenced solely by $x_{t}$. Additionally, $z_{t}$ regulates the extent to which information from $h_{t-1}$ is passed to $h_{t}$.

### 4.2 Long short-term memory (LSTM)

**Fig. 3:**
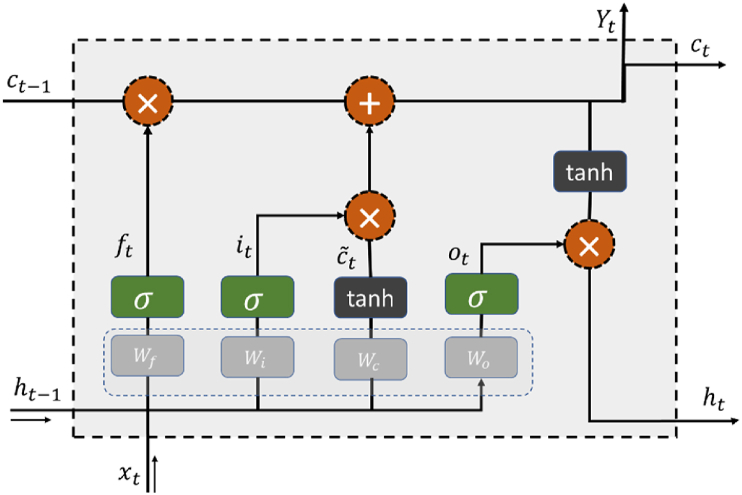
The basic structure of the LSTM unit.

Long Short-Term Memory (LSTM) networks have emerged as a pivotal advancement in the field of neural networks, particularly in the realm of sequence modeling and time series analysis. In response to challenges faced by traditional recurrent neural networks, such as difficulties in learning long-range dependencies, LSTMs introduce a sophisticated architecture with memory cells and gating mechanisms. These components enable LSTMs to selectively store and retrieve information over extended sequences, making them well-suited for tasks that involve temporal dependencies and context preservation. The illustration in Fig. 3 depicts the fundamental configuration of the LSTM unit. In this diagram, $f_{t}$, $i_{t}$, $c_{t}$, and $o_{t}$ denote the forgetting gate, input gate, and cell state, while W and b stand for the associated weight coefficient matrix and bias vector. The output gate is also included, and σ and tanh refer to the sigmoid and hyperbolic tangent activation functions, respectively [32].

To begin, a new input, denoted as $x_{t}^{\prime }$ and formed by combining the external input $x_{t}$ and the previous upper output $h_{t-1}$, is created as $x_{t}^{\prime }=\left[h_{t-1},x_{t}\right]$. This $x_{t}^{\prime }$ is then fed into the forgetting gate, where it undergoes a screening process to filter and retain relevant information, ultimately producing the output $f_{t}$. The details of this screening process are illustrated in Equation below.

(7) $$f_{t}=\sigma \left(W_{f}.\left[h_{t-1},x_{t}\right]+b_{f}\right)$$

At the same time, the input $x_{t}^{\prime }$ is introduced to the input gate, responsible for deciding the information to be stored in the unit state, resulting in the output. The process of updating is depicted in the Equation below:

(8) $$i_{t}=\sigma \left(W_{i}.\left[h_{t-1},x_{t}\right]+b_{i}\right)$$

The activation of $x_{t}^{\prime }$ involves applying the hyperbolic tangent (tanh) function, yielding the output ${\tilde{c}}_{t}$. The activation procedure is illustrated in Formula (9).

(9) $${\tilde{c}}_{t}=\tan\;h\left(W_{c}.\left[h_{t-1},x_{t}\right]+b_{c}\right)$$

Through the joint action of the first three outputs, the state of the memory cell $c_{t-1}$ in the previous stage is updated to state $c_{t}$, and the updating process is shown in Formula (10)

(10) $$c_{t}=f_{t}.c_{t-1}+i_{t}.{\tilde{c}}_{t}$$

The input $x_{t}^{\prime }$ is directed to the output gate, where it influences the state of the output unit. The output $o_{t}$ is derived through Formula (11).

(11) $$o_{t}=\sigma \left(W_{o}.\left[h_{t-1},x_{t}\right]+b_{o}\right)$$

The final output of the LSTM unit denoted as $h_{t}$ or $y_{t}$, is obtained by multiplying the output data $o_{t}$, which has undergone activation through the hyperbolic tangent function, with the new state $c_{t}$. This process is depicted in Formula (12).

(12) $$y_{t}=h_{t}=o_{t}.\tanh\left(c_{t}\right)$$

## 5 Industrial energy hub

In this section, we discuss the details of the proposed energy hub, elucidating its structural components and operational principles. The proposed energy hub is comprised of key elements, including thermal energy storage, a steam generator heat pump, an electric boiler, and seamless integration with a smart grid and a TMP mill. Together, these components synergize to establish a robust and adaptable energy supply system for the paper mill.

### 5.1 Structure of the proposed energy hub

**Fig. 4:**
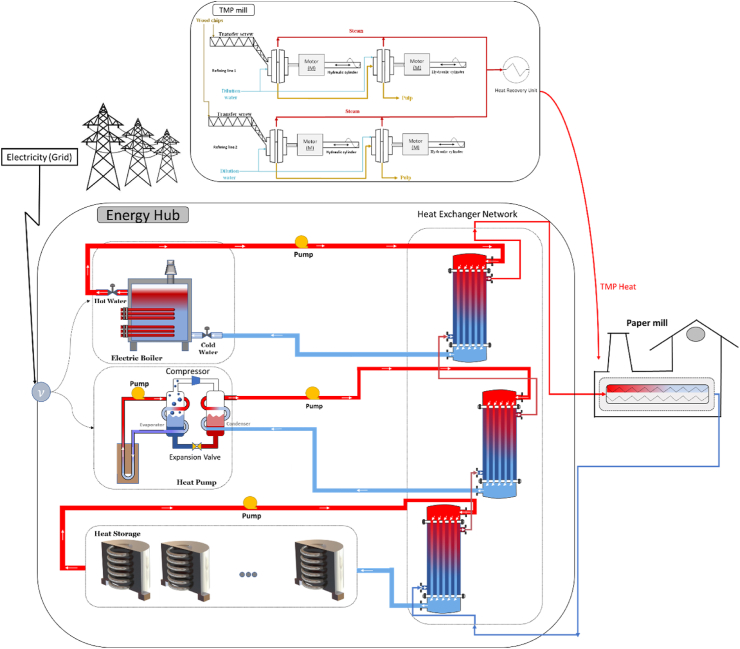
General schematic of the proposed EH system integrated with TMP mill and electricity grid.

The schematic of the Energy Hub, integrated with the paper machine, TMP mill, and electricity grid, is depicted in Fig. 4. The energy hub is responsible for supplying the heat needed for the drying section of the paper machine. Drying is a critical step in papermaking, and the temperature for this process can vary depending on the type of paper being produced and the specific machinery used. Typically, drying temperatures range from 100 °C to 150 °C. The exact temperature may vary based on the type of paper and the machinery used. The assumption in this research is that the temperature required for the drying process in the paper mill is approximately 120°, a temperature close to the fusion temperature of Erythritol. Erythritol has a high fusion temperature, which makes it suitable for the drying section of the paper machine. Additionally, erythritol has a higher heat of fusion, resulting in higher heat density and allowing for a smaller Thermal Energy Storage (TES) system in terms of size. However, the limitation of the TES material (erythritol in our case) is its temperature limit. The fusion temperature of erythritol is just at the border of what can be used to meet the heat demand of the paper machine. To mitigate this risk and ensure that the paper machine consistently operates within the required thermal parameters, it is essential to have an auxiliary heat source. Therefore, an electric boiler should always be on standby. The role of the electric boiler is to provide supplementary heat, ensuring that the temperature is elevated to the desired setpoint whenever the TES material alone cannot meet the demand. This approach guarantees that the process sustainability is not compromised and that there is a reliable and continuous supply of heat to maintain efficient and uninterrupted paper production.

The energy hub incorporates various components, including an electric boiler, a steam heat generator, a heat pump, and thermal energy storage, to efficiently manage and supply the required energy demand. The optimization of thermal energy storage systems is essential for the cost-effective operation of the energy hub. Multiple thermal storage units are employed to ensure full charging, allowing for precise control over the storage rates during operation. The reason for choosing a heat pump and an electric boiler is based on their respective attributes: the heat pump generally has higher energy efficiency compared to the electric boiler (depending on the temperature lift). However, since the heat pump is composed of more components, its overall system reliability is lower, leading to potential unsupplied demand penalty costs. Conversely, the electric boiler has a simpler structure, resulting in higher reliability compared to the heat pump, but its lower efficiency can increase operation costs. The idea behind incorporating both in the energy hub design is their complementary roles, which can help avoid unsupplied demand penalty costs and operation costs. The goal is to minimize the sum of unsupplied demand costs and operation costs, thereby reducing the overall system running costs.

### 5.2 Phase change thermal energy storage

In this research, we employed pure erythritol as the material for Thermal Energy Storage, given its high energy density. Erythritol ($C_{4}H_{10}O_{4}$, ET) is a typical alcohol with a moderate-low temperature range, characterized by its high latent heat and a melting point of 118.8 °C and heat of fusion of 429 (kJ/L). Thanks to its elevated energy storage density, non-toxic nature, resistance to corrosion, and cost-effectiveness, it can serve as a suitable option for heat storage at moderate and lower temperatures. Compared to traditional TES materials like paraffin wax, salt hydrates, and other organic and inorganic compounds, erythritol offers advantages in safety, stability, and environmental impact, though it currently comes at a higher cost. Widely available due to its use in the food industry, erythritol's market dynamics and production scalability could potentially reduce costs over time, making it a viable option for medium-temperature TES applications.

Erythritol used as a phase change material (PCM) in thermal energy storage (TES), typically withstands thousands of thermal cycles before significant degradation, depending on factors like thermal cycling conditions, temperature range, and the presence of impurities or stabilizers. Its melting point of around 118–122 °C and high latent heat of fusion makes it suitable for medium-temperature TES systems. In our case study, the TES system is designed to have a lifespan of 20 years, with an operational cycle of approximately 100 cycles per year. Given this operational context, the material degradation over the system's lifespan is considered to have a negligible impact on our study results. This is due to the fact that the rate of degradation does not significantly influence the system's performance within the specified timeframe and usage pattern. Table 2 presents the primary physical characteristics of erythritol in comparison with water [33].

**Table 2:** Erythritol’s physical characteristics in comparison with water.

Property	Phase	Water	Erythritol
Density $\left(kg/m^{3}\right)$	(Solid)	917 @ 0.0 (°C)	1480 @20 (°C)
	(Liquid)	999.84 @ 0.0 (°C)	1300 @140 (°C)
Specific heat (kJ/kg∙K)	(Solid)	2.101 @ −2.2 (°C)	1.38 @20 (°C)
	(Liquid)	4.217 @ 0.0 (°C)	2.77 @140 (°C)
Thermal conductivity (W/m∙K)	(Solid)	2.09 @ 0.0 (°C)	0.73 @20 (°C)
	(Liquid)	0.565 @ 0.0 (°C)	0.33 @140 (°C)
Melting point (°C)	–	0	119
Heat of fusion	(kJ/kg)	333.6	330
	(kJ/L (liquid))	333.5	429

The energy density of pure erythritol can be calculated using Eq. (13). This calculation allows us to determine the energy stored in erythritol when it transitions from a solid to a liquid state.

(13) $$\text{Energy}\;\text{Density}\;\left(kWh/m^{3}\right)=\text{Heat}\;\text{of}\;\text{Fusion}\;\left(kJ/kg\right)\times \text{Density}\;\left(kg/m^{3}\right)\times \frac{kWh}{3600\;kJ}$$

Erythritol proves ideal for short-term energy storage, capable of efficiently storing energy for a few hours, up to a maximum of one day. Our research operates on the assumption that TES initiates its charging process over a 24-h period as directed by the central hub controller and subsequently discharges the stored energy the following day. Equations (14), (15), (16), (17), (18), (19), (20)) depict the operation constraints and integration of thermal energy storage into the optimal operation of the Energy Hub. ΔT in all the equations below is 1 h.

(14) $$If\sum_{t=i}^{i+24}EP\left(t\right)<\sum_{t=i+24}^{i+48}EP\left(t\right)\;\left(\forall i\in \left\{1,25,49,\ldots ,8737\right\}\right)\to \;I_{\left(i:i+24\right)}=1,J_{\left(i+24:i+48\right)}=1$$

(15) $$If\;I=1\;\left(charging\;mode\right)\to {TES}_{Heat,stored}\left(t=T\right)=\sum_{t=i}^{T}{TES}_{ch,rate}\left(t\right)*\Delta T,\Delta T=1\;hour$$

(16) $$If\;J=1\;\left(discharging\;mode\right)\to {TES}_{Heat,stored}\left(t=T\right)={TES}_{Heat,capacity}^{\max}-\sum_{t=i+24}^{T}{TES}_{dis,rate}\left(t\right)*\Delta T$$

(17) $$If\;I\neq 1\;\&\;J\neq 1\;\left(standby\;mode\right)\to {TES}_{Heat,stored}\left(t\right)=0$$

(18) $${TES}_{Heat,stored}\left(t=24\right)\leq {TES}_{Heat,capacity}^{\max}$$

(19) $${TES}_{ch,rate}^{\min}\leq {TES}_{ch,rate}\left(t\right)\leq {TES}_{ch,rate}^{\max}$$

(20) $${TES}_{dis,rate}^{\min}\leq {TES}_{dis,rate}\left(t\right)\leq {TES}_{dis,rate}^{\max}$$

Equation (14) describes the criteria triggering the initiation of the charging process for thermal energy storage. When the anticipated average electricity price for the following day surpasses that of the current day, the thermal energy storage initiates the charging phase (I=1), with the stored energy intended for discharge on the subsequent day (J=1). Equation (15) explains the stored energy within the Thermal Energy Storage in (MWh) at the time instance t=T, specifically within the ongoing charging process denoted by I=1. In this equation, ${TES}_{ch,rate}\left(t\right)\;\left(MW\right)$ represents the rate at which thermal energy is being charged into the storage system. Equation (16) delineates the stored energy within the Thermal Energy Storage at the time instance t=T, where the discharging condition is denoted by J=1. In this equation, ${TES}_{dis,rate}\left(t\right)$ represents the discharge rate of the thermal energy storage in (MW). Equation (17) guarantees that the stored energy within the thermal energy storage remains at zero during periods when neither charging nor discharging activities are taking place (standby mode). Eq. (18) guarantees that the stored energy stays within a designated range to preserve the integrity of the thermal energy storage system. Equations (19), (20)) set limitations on the charging and discharging rates of the thermal energy storage, aiming to prevent potential overloading.

### 5.3 Cost-efficient EH design and operation

The optimal design of the Energy Hub involves determining the ideal capacities for the energy conversion units. Oversizing can lead to a significant increase in capital costs. The preferred design is to structure the system so that the combined maximum capacity of the energy conversion components aligns with the maximum heat demand of the Energy Hub. Appropriate sizing and optimal allocation of maximum capacity to various components within the energy hub are crucial, as improper sizing may result in system unavailability due to potential Energy Hub component failures, leading to substantial penalties for unsupplied demand for each MWh shortage in heat supply. Equation (21) demonstrates the adaptability in adjusting the maximum capacity for each energy conversion component within the EH. Compliance with the subsequent constraint is imperative for these maximum capacities. ${HP}_{\max,heat}$, ${EB}_{\max,heat}$, and ${HD}_{\max}$ are the heat pump maximum heat production capacity, electric boiler maximum heat production capacity and maximum heat demand of the energy hub respectively.

(21) $${HP}_{\max,heat}+{EB}_{\max,heat}={HD}_{\max}+{TES}_{ch,rate}$$

It is crucial to allocate the correct maximum capacity for energy conversion units, considering variations in the energy efficiency profile, heat demand, and market electricity price over time. Consequently, determining the maximum capacity for components like the heat pump and electric boiler is essential for ensuring system availability and minimizing operational costs. Achieving the optimal design necessitates decision-making grounded in operational costs and system availability. Hence, by considering diverse maximum capacities for the energy hub components, the system attains optimal operational cost and availability. This information is then utilized in subsequent decision-making processes. Equation (22) shows the energy balance within the Energy Hub during charging, discharging, and standby modes. In these equation, ${HP}_{t=T}^{electricity}$ and ${EB}_{t=T}^{electricity}$ are the consumed electricity by steam generator heat pump and electric boiler at the time t=T. ${HD}_{t=T}$ is the heat demand of the Energy hub and ${TES}_{ch,rate}$ is the charging rate of the TES which is considered to be constant value. $\eta_{t=T}^{EB}$ and $\eta_{t=T}^{HP}$ are the efficiencies of the electric boiler and heat pump at the time t=T.

(22) $$If\;I=1\;\left(charging\;mode\right):\eta_{t=T}^{HP}{HP}_{t=T}^{electricity}+\eta_{t=T}^{EB}{EB}_{t=T}^{electricity}={HD}_{t=T}+{TES}_{ch,rate}$$

(23) $$If\;J=1\;\left(discharging\;mode\right):\eta_{t=T}^{HP}{HP}_{t=T}^{electricity}+\eta_{t=T}^{EB}{EB}_{t=T}^{electricity}={HD}_{t=T}-{TES}_{ch,rate}$$

(24) $$If\;I\neq 1\;\&\;J\neq 1\;\left(standby\;mode\right):\eta_{t=T}^{HP}{HP}_{t=T}^{electricity}+\eta_{t=T}^{EB}{EB}_{t=T}^{electricity}={HD}_{t=T}$$

The central hub controller employs optimization techniques to minimize the operational costs of the system at every time step. The objective of the optimization algorithm in each time interval is to minimize the system's electricity costs. The goal is to determine the optimal operating rate for each energy conversion unit relative to its nominal power. Equation (25) illustrates the optimization objective function of the central hub controller operating within each time interval. ${EP}_{t=T}$ is the market electricity price at *t* = *T*.

(25) $$ObjFcn=\min\left[{EP}_{t=T}\left({HP}_{t=T}^{electricity}+{EB}_{t=T}^{electricity}\right)\right]$$

## 6 Continuous Markov process: reliability and availability assessment of the EH

Typically, reliability issues focus on systems that exhibit discrete spatial characteristics, meaning they can occupy various distinguishable states, while also maintaining continuous behavior over time. In other words, they maintain a continuous presence in a specific system state until a transition takes place, moving them discretely to another state where they again persist continuously until the occurrence of another transition. The methods outlined in this section are for systems characterized as stationary Markov processes, meaning that the likelihood of failure or repair during a fixed time interval remains constant. This means that the components' failure and repair behaviors follow negative exponential distributions. For a single component or systems comprising statistically independent components, the steady-state probabilities, or limiting probabilities, are not dependent on the state residence time distributions, only upon their mean values. If the aforementioned conditions are met, the Markov approach can be applied to various reliability problems, encompassing non-repairable and repairable systems as well as those in series, parallel redundancy, or standby redundancy configurations [34].

### 6.1 Evaluating time-dependent state probabilities

**Fig. 5:**
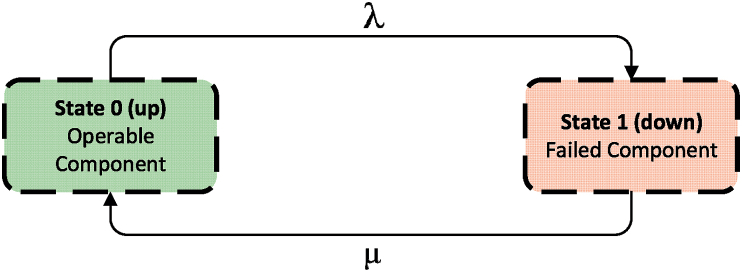
State-space diagram of a repairable system consisting of one component.

The state space diagram specific to the single repairable component can be observed as given in Fig. 5. The transition rate, which includes failure (μ) and repair (λ) rates, represents the shifts between states for a single repairable component. The variable definition for evaluating the system state probabilities is specified as follows:

$$p_{0}\left(t\right)=\text{Probability}\;of\;the\;operable\;component\;at\;time\;t$$

$$p_{1}\left(t\right)=\text{Probability}\;of\;the\;failed\;component\;at\;time\;t$$

$$\mu \;\left(repair\;rate\right)=\frac{number\;of\;repairs\;of\;a\;component\;in\;the\;given\;period\;of\;time}{total\;period\;of\;time\;the\;component\;was\;being\;repaired}$$

$$\lambda \;\left(failure\;rate\right)=\frac{number\;of\;failures\;of\;a\;component\;in\;the\;given\;period\;of\;time}{total\;period\;of\;time\;the\;component\;was\;operating}$$

Now, let's examine a tiny time interval, dt, which has been chosen to be so small that the probability of two or more events taking place within this short duration is extremely low. The probability of remaining in the operational state after the time interval dt – in other words, the probability of being in state 0 as shown in Fig. 5 at time (t + dt) – is:

(26) $$p_{0}\left(t+dt\right)=\left\{\text{probability}\;\text{of}\;\text{zero}\;\text{failures}\;\text{in}\;\text{the}\;\text{interval}\;\left(0,t\right)\times \text{probability}\;\text{of}\;\text{zero}\;\text{failures}\;\text{in}\;\text{the}\;\text{interval}\;\left(t,t+\text{dt}\right)\right\}+\left\{\text{probability}\;\text{of}\;\text{being}\;\text{failed}\;\text{in}\;\text{the}\;\text{interval}\;\left(0,t\right)\times \text{probability}\;\text{of}\;\text{being}\;\text{repaired}\;\text{in}\;\text{time}\left(t,t+\text{dt}\right)\right\}$$

The mathematical representation of Eq. (26) is given in Eq. 27.a.

(27.a) $$p_{0}\left(t+dt\right)=p_{0}\left(t\right)\left(1-\lambda dt\right)+p_{1}\left(t\right)\left(\mu dt\right)$$

Similarly for state 1:

(27.b) $$p_{1}\left(t+dt\right)=p_{0}\left(t\right)\left(1-\lambda dt\right)+p_{1}\left(t\right)\left(\mu dt\right)$$

from Equation 27.a:

(28) $$\frac{p_{0}\left(t+\text{dt}\right)‐p_{0}\left(t\right)}{\text{dt}}=‐\lambda p_{0}\left(t\right)+\mu p_{1}\left(t\right)\;\text{as}\;\text{dt}\to 0$$

(29) $${\frac{p_{0}\left(t+dt\right)-p_{0}\left(t\right)}{dt}|}_{dt\to 0}=\frac{dp_{0}\left(t\right)}{dt}=p_{0}^{\prime }\left(t\right)\;\text{thus,}$$

(30.a) $$p_{0}^{\prime }\left(t\right)=-\lambda p_{0}\left(t\right)+\mu p_{1}\left(t\right)$$

(30.b) $$p_{1}^{\prime }\left(t\right)=\lambda p_{0}\left(t\right)-\mu p_{1}\left(t\right)$$

Equation (30.a), (30.b) are linear differential equations with constant coefficients. Various methods are available to solve these equations, but one of the simplest and commonly employed techniques is the utilization of Laplace transforms. For instance, the Laplace transformation of this equation is as follows:

(31) $${sp}_{0}\left(s\right)-p_{0}\left(0\right)=-\lambda p_{0}\left(s\right)+\mu p_{1}\left(s\right)$$

where $p_{i}\left(s\right)$ is the Laplace transform of $p_{i}\left(t\right)$ and $p_{0}\left(0\right)$ is the initial value of $p_{0}\left(t\right)$. Rearranging Equation (31) gives:

(32.a) $$p_{0}\left(s\right)=\frac{\mu }{s+\lambda }p_{1}\left(s\right)+\frac{1}{s+\lambda }p_{0}\left(0\right)$$

Similarly Equation 30. b can be transformed into:

(32.b) $$p_{1}\left(s\right)=\frac{\lambda }{s+\mu }p_{0}\left(s\right)+\frac{1}{s+\mu }p_{1}\left(0\right)$$

Equation (32.a), (32.b) can be solved for $p_{0}\left(s\right)$ and $p_{1}\left(s\right)$ as a system of linear equations either through a direct substitution method or by applying matrix-based solution techniques.

(33.a) $$p_{0}\left(s\right)=\frac{\mu }{\lambda +\mu }\left[\frac{p_{0}\left(0\right)+p_{1}\left(0\right)}{s}\right]+\frac{1}{\lambda +\mu }.\frac{1}{s+\lambda +\mu }\left[\lambda p_{0}\left(0\right)-\mu p_{1}\left(0\right)\right]$$

(33.b) $$p_{1}\left(s\right)=\frac{\lambda }{\lambda +\mu }\left[\frac{p_{0}\left(0\right)+p_{1}\left(0\right)}{s}\right]+\frac{1}{\lambda +\mu }.\frac{1}{s+\lambda +\mu }\left[\mu p_{1}\left(0\right)-\lambda p_{0}\left(0\right)\right]$$

Equation (33.a), (33.b) need to be converted back into the time domain by applying inverse Laplace transforms. The inverse transform of 1/s is 1 and 1/(s+a) is $e^{-at}$, which gives:

(34.a) $$p_{0}\left(t\right)=\frac{\mu }{\lambda +\mu }\left[p_{0}\left(0\right)+p_{1}\left(0\right)\right]+\frac{e^{-\left(\lambda +\mu \right)t}}{\lambda +\mu }\left[\lambda p_{0}\left(0\right)-\mu p_{1}\left(0\right)\right]$$

(34.b) $$p_{1}\left(t\right)=\frac{\lambda }{\lambda +\mu }\left[p_{0}\left(0\right)+p_{1}\left(0\right)\right]+\frac{e^{-\left(\lambda +\mu \right)t}}{\lambda +\mu }\left[\mu p_{1}\left(0\right)-\lambda p_{0}\left(0\right)\right]$$

The term $p_{0}\left(0\right)+p_{1}\left(0\right)=1$ for all initial conditions. In practice the most likely state in which the system starts is state 0, i.e. the system is in an operable condition at zero time ($p_{0}\left(0\right)=1,p_{1}\left(0\right)=0$). Equation (34.a), (34.b) reduce to the frequently quoted equations for the time-dependent probabilities of a single repairable component given by:

(35.a) $$p_{0}\left(t\right)=\frac{\mu }{\lambda +\mu }+\frac{\lambda e^{-\left(\lambda +\mu \right)t}}{\lambda +\mu }$$

(35.b) $$p_{1}\left(t\right)=\frac{\lambda }{\lambda +\mu }-\frac{\lambda e^{-\left(\lambda +\mu \right)t}}{\lambda +\mu }$$

The probabilities $p_{0}\left(t\right)$ and $p_{1}\left(t\right)$ are the probabilities of being found in the operating state and failed state respectively as a function of time given that the system started at time t=0 in the operating state. To assess limiting probabilities or steady-state probabilities, you can examine Equation (8) by allowing t to approach infinity in Equation (8), limiting probabilities or steady-state probabilities of a single repairable component can be achieved. The limiting state probabilities of a system with single repairable component is given below:

(36.a) $$p_{0}\left(t\right)=\frac{\mu }{\lambda +\mu }$$

(36.b) $$p_{1}\left(t\right)=\frac{\lambda }{\lambda +\mu }$$

**Fig. 6:**
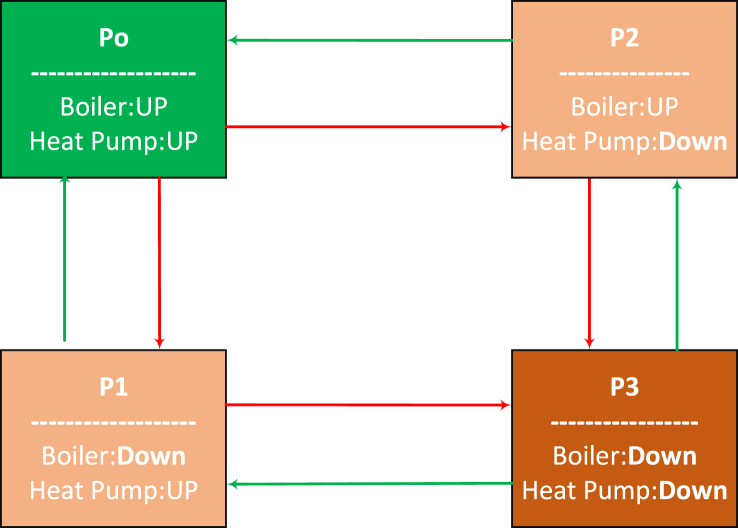
State-space diagram of the heat supply system consisting of energy hub and TMP mill.

If we employ a similar method to compute the time-dependent probabilities of a system comprising two repairable components, the limiting state probabilities will converge as described in Eq. 37.a - Eq. 37.d. The system's state space diagram, which includes an electric boiler and a heat pump, is illustrated in Fig. 6. The system is denoted as ‘up’ when it is functioning, while a non-operational system is characterized as being in a ‘down’ state.

(37.a) $$p_{0}\left(t\to \infty \right)=\frac{\mu_{1}\mu_{2}}{\left(\lambda_{1}+\mu_{1}\right)\left(\lambda_{2}+\mu_{2}\right)}$$

(37.b) $$p_{1}\left(t\to \infty \right)=\frac{\lambda_{1}\mu_{2}}{\left(\lambda_{1}+\mu_{1}\right)\left(\lambda_{2}+\mu_{2}\right)}$$

(37.c) $$p_{2}\left(t\to \infty \right)=\frac{\mu_{1}\lambda_{2}}{\left(\lambda_{1}+\mu_{1}\right)\left(\lambda_{2}+\mu_{2}\right)}$$

(37.d) $$p_{3}\left(t\to \infty \right)=\frac{\lambda_{1}\lambda_{2}}{\left(\lambda_{1}+\mu_{1}\right)\left(\lambda_{2}+\mu_{2}\right)}$$

### 6.2 Failure and the repair rate of series systems

In this section, the failure and repair rates of the system composed of a series of components are addressed. Reference [35] presents a methodology for calculating the failure and repair rates of a series system with a consistent transition rate. The equations presented below provide the procedure for determining the failure and repair rates of a system composed of N-series components.

(38) $$\lambda_{N}=\sum_{j=1}^{N}\lambda_{j}$$

(39) $$\mu_{N}=\frac{\sum_{j=1}^{N}\lambda_{j}}{\sum_{j=1}^{N}\lambda_{j}/\mu_{j}}$$

where N is the number of series components, and j determines each subsystem component. The heat pump consists of a series of five components more likely to fail: compressor, evaporator, condenser, expansion valve, and heat recovery steam generator. The failure and repair rate of the whole heat pump can be calculated by substituting each subsystem's failure and repair rate in Eq. (38) and Eq. (39), respectively.

## 7 Steam generator heat pump (SGHP)

30 % of the overall energy production is attributed to industrial energy consumption, with over half of this energy dedicated to steam generation at different temperatures. Consequently, diverse technologies for heat production are being advanced to minimize the energy needed for industrial steam generation. Notably, approximately 50 % of the total industrial waste heat falls within the temperature range of 60–100 °C. However, it is imperative to cool discharged hot water to below 40 °C to mitigate environmental impact. Consequently, the development of industrial waste heat recovery is crucial for curtailing primary industrial energy consumption [36].

**Fig. 7:**
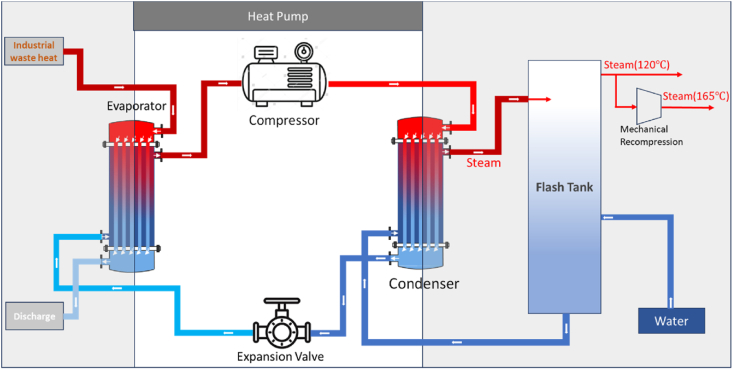
Operating principle of industrial steam generator heat pump system.

The utilization of a steam generator heat pump (SGHP) is widespread for transforming low-temperature waste heat into high-temperature steam. This technology proves effective in recovering waste heat from various industries, generating steam for applications such as heating, drying, humidification, and other industrial processes requiring heat. A vapor compression heat pump, employing electricity in a compressor to transfer heat from lower to higher temperatures, constitutes the core of the SGHP system. The schematic representation of an industrial steam generator heat pump system is illustrated in Fig. 7. In this study, the waste heat emanating from the paper machine (typically ranging from 40 to 60 °C) could potentially serve as low-temperature waste heat for the heat pump evaporator, thereby enhancing overall efficiency by reducing the lifting temperature. However, due to unavailability of specific data on the amount of waste heat from the paper machine, this aspect is not considered in our analysis, and we assume the evaporator temperature to be equivalent to the external temperature.

CO2, also known as R744, stands out as an environmentally friendly refrigerant boasting impeccable thermodynamic properties suitable for a myriad of applications. Its distinction lies in a low critical temperature and elevated operating pressure, setting it apart from other refrigerants. Despite its non-flammable nature and low toxicity, R744 operates under high pressure, necessitating a specialized system design that enhances complexity. While the refrigerant cost remains low, the overall system cost surpasses that of conventional systems. The primary potential risk associated with employing CO2 as a refrigerant is the formation of dry ice, particularly when pressure and temperature drop below the triple point (4.2 bar, −56 °C).

The Coefficient of Performance (COP) of a heat pump can be calculated by first determining the Carnot COP, which is the theoretical maximum efficiency derived from the Carnot cycle. The Carnot COP for heating is given by:

(40) $$COP_{Carnot}={\;T}_{h}\;/\;\left({\;T}_{h}-\;{\;T}_{c}\right)$$

where ${\;T}_{h}$ is the absolute temperature of the hot reservoir and ${\;T}_{c}$ is the absolute temperature of the cold reservoir. The actual COP of the heat pump is typically lower due to real-world inefficiencies and can be calculated as:

(41) $$COP_{actual}=\eta_{Carnot\;}\times COP_{Carnot}$$

where $\eta_{Carnot\;}$ is the coefficient of Carnot efficiency, representing the ratio of the actual performance to the ideal Carnot performance. This coefficient accounts for the losses and imperfections in the system.

## 8 Results and discussion

To compute the system's availability, it is necessary to determine the steady state probabilities of the system within the state-space diagram. In Chapter 3, the process of determining time-dependent and limiting state probabilities for a system comprised of repairable components is discussed. The final results, showing the steady state probabilities, are presented in Table 3.

**Table 3:** Limiting state probabilities of the entire heat supply system.

States	Steady state Probabilities	States	Steady states Probabilities
$s_{0}$	0.950	$s_{2}$	0.035
$s_{1}$	0.015	$s_{3}$	0.0005

The precise availability of the EH system must be calculated according to the EH's demand. The Markov method proposes the steady probability of State 0, where all system components are operational, as the system's availability. However, this approach may not be suitable for the studied energy hub because the EH system could be considered as available system when it can fulfill the hourly demand, irrespective of whether all system components are operational. For example, there are instances where the EH can completely fulfill the demand even if a single component, such as the heat pump, has failed. This is because the demand is lower than the capacity of the electric boiler. In such scenarios, the EH could be deemed available, even if not all system components are operational. Equation (42) shows the actual availability of the system (SA) based on the Energy Hub demand profile. This concept has been previously introduced in Ref. [34].

(42) $$SA=\sum_{i=0}^{3}p_{i}{SA}_{state=i}$$

where ${SA}_{state}$ represents the system availability at each state based on the demand-supply profile, and i denotes the state of the system corresponding to the state space diagram. The system availability at each state can be determined using the following equation. For the calculation, we employ the step function *u*, wherein the function yields an output of 1 when the heat supply capacity (HSC) exceeds the heat demand (HD), and produces an output of 0 when the heat supply capacity is less than the heat demand.

(43) $${SA}_{state=i}=\frac{1}{T}\sum_{t=1}^{t=T}u\left({HSC}_{t,i}-{HD}_{t}\right),T=8765\;h$$

### 8.1 Results of TES design and economic analysis

The primary expense in thermal energy storage is associated with the phase change material (PCM) utilized to supply latent heat, and Chapter 5.2 addresses the methodology employed to calculate the size of the thermal energy storage. Table 4 presents the relevant information and outcomes concerning the design of TES for the proposed EH system, where Equation (13) is utilized to calculate the energy density. The considered thermal energy storage system is intended to possess a capacity of 16.8 MW-hours, featuring a charge and discharge rate of 0.7 MW. This design is based on the demand load of the energy hub, which is determined by the heat demand of the paper machine and the heat production from the thermomechanical pulp mill. The charging rates considered in this design are assumptions that are intended to align with the system's structure and feasibility. Specifically, the assumption is made that the thermal energy storage (TES) released heat rate should be less than the minimum heat load of the energy hub. This ensures that we do not face the issue of excess heat being stored in the TES when there is no demand for it. By employing Table 2 and Equation (13) for determining the energy density of the thermal energy storage, the size of the TES as a singular unit will be computed as $95\;m^{3}$. Given the considerable size of the storage tank, issues arise in terms of both placement and manufacturing, as well as the charging/discharging process due to the substantial mass of the material. To address these challenges, it has been proposed to have three TES units, aiming to minimize the size of each individual unit.

**Table 4:** Technical and cost traits associated with individual thermal energy storage (TES) units.

TES System	Values	Unit
Material	Erythritol	–
Material cost (per kg)	0.47	€/kg
Charge/Discharge rate	0.7	MW
Maximum Capacity	16.8	MWh
PCM material size for the whole TES units	95	$m^{3}$
PCM Material size for each TES unit	31.6	$m^{3}$
Total Material cost	58k	€

### 8.2 Economic viability of the suggested energy hubs: cost and availability analysis

This study explores the viability of incorporating Thermal Energy Storage within an industrial Energy Hub, aiming to closely replicate real-world TES operations in our modeling approach. The study utilizes the years 2021 and 2022 as the reference period for the analysis, comparing the obtained results to assess the robustness of the proposed energy hub system. As outlined in section 5.2, which explores into the modeling approach of the TES system within the Energy Hub configuration, the initiation of the charging process for Thermal Energy Storage occurs when the total hourly electricity price for the upcoming day surpasses the cumulative electricity price for the current day.

The Energy Hub includes energy conversion components, namely an electric boiler and a heat pump. While heat pumps exhibit high efficiency, with a coefficient of performance (COP) reaching up to 2.5 depending on the temperature lift, their limitation lies in the production of high-temperature heat. Moreover, compared to electric boilers, heat pumps face challenges related to reliability due to their more complex structure and a higher number of components. Therefore, the energy conversion components of the Energy Hub encompass both an electric boiler and a heat pump, aiming to guarantee efficient heat production, optimal temperature control, and heightened reliability and availability for the system.

**Fig. 8:**
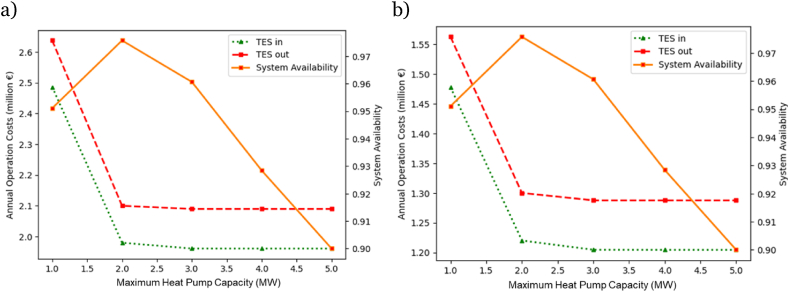
System availability and annual operation cost for the cases where TES is included (TES in) and not included (TES out) in the EH design. a) reference year 2022, b) reference year 2021.

**Fig. 9:**
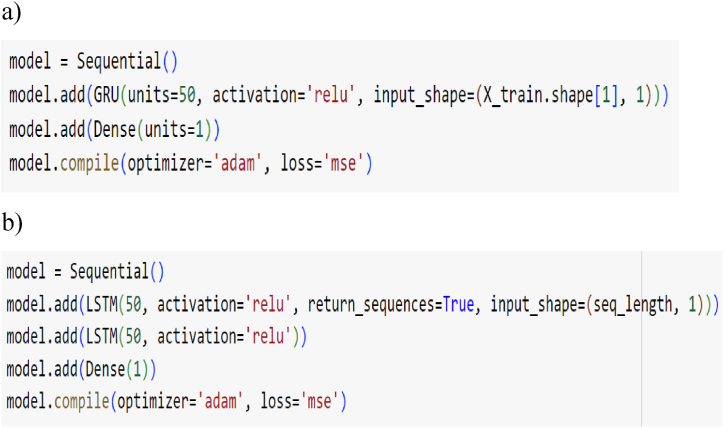
The structure of the studied RNN-like time series model: a) GRU, b) Stacked-LSTM.

Fig. 8 depicts the variations in the Energy Hub's annual operation cost (indicated on the left y-axis) depending on the maximum heat pump capacity in the EH system. Furthermore, the same figure presents the Energy Hub's availability in terms of providing the heat demand, represented on the right y-axis, based on the maximum capacity of the heat pump in the EH system. The calculation method of system availability is given in Formula 35 and 36. A higher capacity for the heat pump leads to decreased operational costs, driven by its superior energy conversion efficiency compared to the electric boiler. Nonetheless, the essential integration of the electric boiler becomes imperative to ensure the consistent supply of the desired temperature and maintain high system availability. This dual-component approach aims to balance efficiency gains with the specific temperature control and reliability requirements of the system. After establishing the maximum capacity of the heat pump using Fig. 9, the calculation for the maximum capacity of the electric boiler in the Energy Hub configuration will involve deducting the maximum heat load of the Energy Hub from the heat pump's capacity. Selecting the ideal capacity for the energy conversion units within the Energy Hub setting is essential for maximizing system availability, thereby minimizing the cost associated with unmet heat demand, enhancing system robustness, and reducing overall system operational costs.

In the analysis of the annual operation cost, it is important to note that the cost of an unsupplied demand penalty is not included in the operation cost. This omission explains why the annual operation cost appears to reduce slightly when deviating from the optimal point by increasing the share of the heat pump. While it is true that the annual operation cost for running the system decreases because heat pumps generally exhibit higher efficiency than electric boilers in heat generation, the overall reliability and availability of the system are compromised when the heat pump capacity is increased at the expense of the electric boiler capacity. This reduction in reliability means that the system is less capable of meeting the heat demand, potentially imposing significant costs on the energy hub operator. The penalty cost for each kWh shortage in meeting the demand could be 15–20 times higher than the selling price to the customer, thereby significantly impacting the economic viability of the system. In this study, the assumption is that the Thermal Energy Storage (TES) is integrated into the system where the system is at the optimal design point. Therefore, the focus of this study is not on the amount of savings from unsupplied demand penalty costs. These issues have been addressed in our previous studies, which can be found in Refs. [2,34].

The depicted results in Fig. 8 emphasize the resilience and effectiveness of the proposed energy hub design strategy, irrespective of the reference year chosen for the study. In both reference years, the annual operational costs have decreased, and the system availability has reached its peak at the optimal design point where the capacity of the steam generator heat pump is considered to be 2 MW. The optimum capacity of 2 MW for a heat pump corresponds to 3.4 MW maximum size capacity for an electric boiler, based on the calculation derived from Eq. (21). Therefore, the optimal corresponding capacity of each energy conversion component can be calculated from Eq. (21) if we know the optimal capacity of the other one. This relationship allows for precise determination of system components, ensuring that each part operates at its highest efficiency.

As a decrease in system availability leads to a substantial increase in the unsupplied demand penalty cost, the optimal design is planned to prioritize maximum system availability. The unsupplied demand penalty cost represents the running cost of the system, which will be imposed on the energy hub operator in addition to the system operation cost. Hence, the selection of a 2 MW power capacity for the heat pump is justified as part of the optimal design for the Energy Hub. Unsupplied demand penalty cost refers to the financial consequences incurred when a system fails to meet or supply the demanded energy. For instance in the studied EH, if the system availability is reduced, and as a result, it fails to meet the paper machine heat demand, there would be a penalty cost (15–20 times greater than the quantity of heat units sold [2]) associated with the unfulfilled or unsupplied demand. This penalty cost is incurred due to the negative impact on the reliability and performance of the system, leading to financial consequences or penalties for failing to meet expected energy demands.

Table 5 provides a cost evaluation concerning the implementation of Thermal Energy Storage within the proposed energy hub system. The study examines the yearly advantages derived from reducing the overall operational costs of the system when employing TES technology (case 2). It then contrasts this scenario with the situation where TES is not incorporated into the energy hub system (case 1). The outcomes correspond to the optimal design point of the energy hub system, as determined from Fig. 8. At this optimal design point, the operational cost is comparatively minimized, while the system's availability is maximized. From Table 5, both scenarios demonstrate a consistent system availability of 98 %, independent of the TES system, as it is dependent upon the maximum capacity of energy conversion units in the EH setup. The influence of the TES system on system availability is minimal, given its capacity is significantly lower than the heat demand.

**Table 5:** Cost and availability analysis of the energy hub (where the system availability is 98 %).

Reference study year	Cost (€), optimal design point	Case 1 (TES out)	Case 2 (TES in)	Changes (Case 1- Case 2)	% Differences w.r.t case 1
2022					
	Annual operation cost	2.1 (M€)	1.98 (M€)	−120 (K€)	−5.71
	Total operation cost (life span:20 years)	37.8 (M€)	35.64 (M€)	−2.16 (M€)	−5.71
	TES material capital cost	–	58 (K€)	–	–
	System availability	98 %	98 %	–	–
2021					
	Annual operation cost	1.3 (M€)	1.22 (M€)	−80 (K€)	−4.36
	Total operation cost (life span:20 years)	23.40 (M€)	21.96 (M€)	−1.44 (M €)	−4.36
	TES material capital cost	–	58 (K€)	–	–
	System availability	98 %	98 %	–	–

The TES material capital cost amounts to 58k euros. When put beside with the overall reduction in the system total operation cost over the system lifespan, the percentage of the TES capital cost is equal to 2.68 % (year 2022) and 4.02 % (year 2021) of the financial advantage associated with the thermal energy storage, underscoring the substantial impact of the investment.

### 8.3 Market electricity price prediction

In this section, we explore the outcomes of employing various LSTM models to predict electricity prices. Given the critical importance of understanding the average daily electricity price for the day ahead in the context of charging and discharging heat storage, it becomes imperative to create a model for forecasting this key parameter. This predictive model is essential for evaluating the cost-effectiveness of charging the heat storage system. The ability to anticipate the day-ahead average daily electricity price is fundamental to assessing the economic viability of utilizing heat storage in coordination with electricity pricing dynamics. The structure of both time series models is depicted in Fig. 9, which details how the model's structure is employed in this research and its implementation in the Python code.

The Stacked-LSTM model consist of a sequential neural network model using the Keras library. The model consists of two Long Short-Term Memory layers with 50 units each and Rectified Linear Unit (ReLU) activation function. The model is then followed by a Dense layer with 1 unit. The model is compiled using the Adam optimizer and Mean Squared Error as the loss function, commonly used for regression tasks. However the GRU model comprise of a neural network model with a Gated Recurrent Unit layer having 50 units and ReLU activation. The model concludes with a Dense layer having 1 unit.

**Fig. 10:**
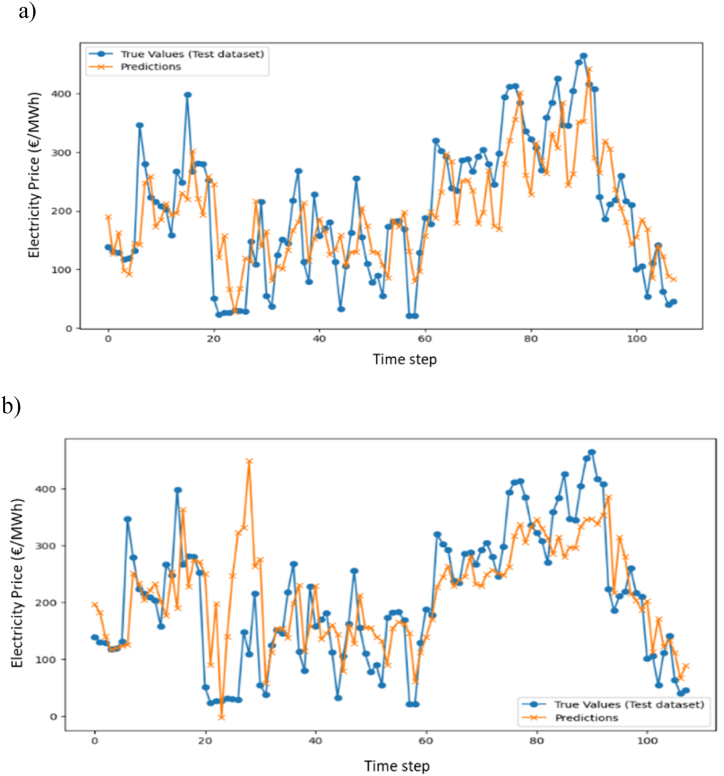
Actual and predicted average daily electricity prices on the test dataset: a) GRU, b) Stacked-LSTM.

Fig. 10 illustrates the actual and predicted average daily electricity prices on the test dataset, showcasing the outcomes of developing a time series model with various last structures. The proximity between the predicted and actual daily average electricity prices at each time step serves as an indicator of the accuracy of the prediction method.

Detailed metrics assessing the accuracy of each LSTM-like model are presented in the Table 6. For the GRU model, the RMSE is 67.24 on the train set and 75.79 on the test set. The corresponding R-squared values are 0.82 and 0.72, respectively. In comparison, the Stacked LSTM model has an RMSE of 69.09 on the train set and 90.97 on the test set, with R-squared values of 0.77 and 0.61. Comparatively, for both the train and test datasets, the GRU model has lower RMSE values, indicating better performance in terms of predictive accuracy. Additionally, the R-squared values are higher for the GRU model on both datasets, suggesting a better fit to the data compared to the Stacked LSTM model.

**Table 6:** Accuracy results for the studied time series models: GRU and Stacked-LSTM.

Model	Train dataset		Test dataset	
	RMSE	R	RMSE	R
GRU	67.24	0.82	75.79	0.72
Stacked LSTM	69.09	0.77	90.97	0.61

## 9 Conclusion

Thermal Energy Storage (TES) emerges as a practical solution for cutting industrial operational expenses by efficiently redistributing heat demands within industrial processes. In the integrated Thermomechanical pulping process, TES systems can be seamlessly integrated into the energy hub to provide the heat requirements of the paper machine. The principal aim of incorporating TES within the energy hub is to minimize electricity costs, particularly during peak hours. This strategic application of TES technology fosters a more economical and streamlined management of energy consumption within the energy hub, ultimately contributing to overall operational savings for the system. This research introduces an innovative approach to optimizing the design and operation of an Energy Hub with Thermal Energy Storage in the forest industry. The proposed method involves a thorough analysis of the dynamic efficiency, reliability, and availability of system components to achieve optimal design. The Energy Hub features energy conversion technologies such as an electric boiler and a steam generator heat pump. The study explores the impact of system reliability on operational costs and evaluates how the maximum capacities of the components influence this reliability. The method determines the optimal design point to maximize system reliability benefits. Additionally, to optimize the TES system's charging and discharging schedule, an advanced predictive method using time series prediction models, including Long Short-Term Memory (LSTM) and Gated Recurrent Unit (GRU), has been developed to forecast average daily electricity prices.

The findings indicate that the most efficient configuration for the proposed energy hub for the forest industry application necessitates a steam generator heat pump with a maximum capacity of 2 MW and an electric boiler with a capacity of 3.4 MW. In this optimized EH configuration, the system availability achieves a peak of 98 percent, accompanied by a notably elevated reduction in operational costs when integrating TES into the EH. In the quest for an optimal schedule for TES charging and discharging, the GRU method has proven to outperform LSTM in accurately predicting electricity prices. This underscores its potential as a reliable and accurate model for forecasting electricity prices within the system. The study compares the costs and system availability of energy systems with and without Thermal Energy Storage (TES) for 2021 and 2022. In both years, incorporating TES reduces the annual and total operation costs by 5.71 % and 4.36 %, respectively, while maintaining system availability at 98 %. The TES material capital cost of 58k euros represents 2.68 % (2022) and 4.02 % (2021) of the overall system operation cost reduction over its lifespan, highlighting the significant financial impact of the investment.

## 10 Future work suggestion

Future work in the continuation of this study will focus on developing a model to predict the dynamic COP value of the heat pump for optimal operation of the energy hub in integration with the thermal energy storage (TES). Considering the presence of the heat pump, it is crucial to account for the dynamic changes in its efficiency based on temperature lift within the optimization model. For example, depending on the electricity price profile and heat load profile of the energy hub, the model should determine the appropriate technology to charge the TES. Neglecting this consideration could lead to inefficient system operation in terms of cost during certain charging/discharging cycles.

## Funding

This project has received funding from the European Union – NextGenerationEU instrument and is funded by the 10.13039/501100002341Research Council of Finland under grant number 353299.

## Data availability statement

The data for this study is confidential and not available in a public repository.

## CRediT authorship contribution statement

**Behnam Talebjedi:** Writing – review & editing, Writing – original draft, Visualization, Validation, Supervision, Software, Resources, Methodology, Investigation, Formal analysis, Data curation, Conceptualization. **Timo Laukkanen:** Writing – review & editing, Supervision, Project administration, Investigation, Funding acquisition, Conceptualization. **Henrik Holmberg:** Writing – review & editing, Supervision, Project administration, Investigation, Funding acquisition, Conceptualization.

## Declaration of competing interest

The authors declare that they have no known competing financial interests or personal relationships that could have appeared to influence the work reported in this paper.

## References

[1] Talebjedi B., Laukkanen T., Holmberg H., Vakkilainen E., Syri S. (Mar. 2021). Energy efficiency analysis of the refining unit in thermo-mechanical pulp mill. Energies.

[2] Talebjedi B., Laukkanen T., Holmberg H., Syri S. (2023). Advanced design and operation of Energy Hub for forest industry using reliability assessment. Appl. Therm. Eng..

[3] Qu X., Qi X., Zhang Y., Zhou D. (Jul. 2024). Performance of a rotating latent heat thermal energy storage unit with heat transfer from different surfaces. Appl. Therm. Eng..

[4] Tawalbeh M., Khan H.A., Al-Othman A., Almomani F., Ajith S. (May 2023). A comprehensive review on the recent advances in materials for thermal energy storage applications. International Journal of Thermofluids.

[5] Miró L., Gasia J., Cabeza L.F. (Oct. 01, 2016). Thermal energy storage (TES) for industrial waste heat (IWH) recovery: a review. Appl. Energy.

[6] Opolot M., Zhao C., Liu M., Mancin S., Bruno F., Hooman K. (2022). A review of high temperature (≥ 500 °C) latent heat thermal energy storage. Renew. Sustain. Energy Rev..

[7] Davoudi M., Moeini-Aghtaie M., Ghorani R. (Jul. 2021). Developing a new framework for transactive peer-to-peer thermal energy market. IET Gener., Transm. Distrib..

[8] Calderón A., Barreneche C., Hernández-Valle K., Galindo E., Segarra M., Fernández A.I. (Apr. 2020). Where is Thermal Energy Storage (TES) research going? – a bibliometric analysis. Sol. Energy.

[9] Reddy K.S., Mudgal V., Mallick T.K. (Feb. 01, 2018). Review of latent heat thermal energy storage for improved material stability and effective load management. J. Energy Storage.

[10] Cirocco L. (2022). Thermal energy storage for industrial thermal loads and electricity demand side management. Energy Convers. Manag..

[11] Barnetche M., González-Portillo L.F., Abbas R. (2023). Optimum integration of latent heat storage in a solar thermal system for industrial processes: in series or in parallel?. Appl. Therm. Eng..

[12] Hengrui M. (Nov. 2023). An effective planning approach for integrated energy systems considering equipment operating characteristics. Heliyon.

[13] Ben Amor M., Billette de Villemeur E., Pellat M., Pineau P.O. (Mar. 2014). Influence of wind power on hourly electricity prices and GHG (greenhouse gas) emissions: evidence that congestion matters from Ontario zonal data. Energy.

[14] Kumar Sharma D., Prakash Varshney R., Agarwal S., Ali Alhussan A., Abdallah H.A. (Apr. 2024). Developing a multivariate time series forecasting framework based on stacked autoencoders and multi-phase feature. Heliyon.

[15] Siddique M.A.B. (Mar. 2024). Forecasting of tilapia (Oreochromis niloticus) production in Bangladesh using ARIMA model. Heliyon.

[16] Tan Z., Zhang J., Wang J., Xu J. (2010). Day-ahead electricity price forecasting using wavelet transform combined with ARIMA and GARCH models. Appl. Energy.

[17] Yan X., Chowdhury N.A. (2013). Mid-term electricity market clearing price forecasting: a hybrid LSSVM and ARMAX approach. Int. J. Electr. Power Energy Syst..

[18] Singhal D., Swarup K.S. (Mar. 2011). Electricity price forecasting using artificial neural networks. Int. J. Electr. Power Energy Syst..

[19] Memarzadeh G., Keynia F. (2021). Short-term electricity load and price forecasting by a new optimal LSTM-NN based prediction algorithm. Elec. Power Syst. Res..

[20] Coelho C., Costa M.F.P., Ferrás L.L. (2024). Enhancing continuous time series modelling with a latent ODE-LSTM approach. Appl. Math. Comput..

[21] Xiong X., Qing G. (2023). A hybrid day-ahead electricity price forecasting framework based on time series. Energy.

[22] Talebjedi B., Laukkanen T., Holmberg H., Vakkilainen E., Syri S. (Sep. 2022). Advanced energy-saving optimization strategy in thermo-mechanical pulping by machine learning approach. Nord. Pulp Pap Res. J..

[23] Bouktif S., Fiaz A., Ouni A., Serhani M.A. (2018). Optimal deep learning LSTM model for electric load forecasting using feature selection and genetic algorithm: comparison with machine learning approaches. Energies.

[24] Taheri S., Talebjedi B., Laukkanen T. (2021). Electricity demand time series forecasting based on empirical mode decomposition and long short-term memory. Energy Eng. J. Assoc. Energy Eng.: Journal of the Association of Energy Engineering.

[25] Xiong B. (2023). A flow-rate-aware data-driven model of vanadium redox flow battery based on gated recurrent unit neural network. J. Energy Storage.

[26] Sarbu I., Sebarchievici C. (2018). A comprehensive review of thermal energy storage. Sustainability.

[27] Kalidasan B., Pandey A.K., Saidur R., Han T.K., Mishra Y.N. (2024). MXene-based eutectic salt hydrate phase change material for efficient thermal features, corrosion resistance & photo-thermal energy conversion. Materials Today Sustainability.

[28] Kalidasan B., Pandey A.K., Aljafari B., Chinnasamy S., Kareri T., Rahman S. (Dec. 2023). Thermo-kinetic behaviour of green synthesized nanomaterial enhanced organic phase change material: model fitting approach. J. Environ. Manag..

[29] Tian Y., Zhao C.Y. (2013). A review of solar collectors and thermal energy storage in solar thermal applications. Appl. Energy.

[30] Ghosh A.K. (2011). Evaporation, Condensation and Heat Transfer.

[31] Chen Y., Liu G., Huang X., Chen K., Hou J., Zhou J. (Jul. 2021). Development of a surrogate method of groundwater modeling using gated recurrent unit to improve the efficiency of parameter auto-calibration and global sensitivity analysis. J. Hydrol. (Amst.).

[32] Song Y., Cai C., Ma D., Li C. (2024). Modelling and forecasting high-frequency data with jumps based on a hybrid nonparametric regression and LSTM model. Expert Syst. Appl..

[33] Hirano S. (2012).

[34] Talebjedi B., Behbahaninia A. (2021). Availability analysis of an Energy Hub with CCHP system for economical design in terms of Energy Hub operator. J. Build. Eng..

[35] Wang J.J., Fu C., Yang K., Zhang X.T., hua Shi G., Zhai J. (2013). Reliability and availability analysis of redundant BCHP (building cooling, heating and power) system. Energy.

[36] Kang D.H., Na S.I., Kim M.S. (2017). Recent researches on steam generation heat pump system. International Journal of Air-Conditioning and Refrigeration.

